# Mutual chemical effect of autograft and octacalcium phosphate implantation on enhancing intramembranous bone regeneration

**DOI:** 10.1080/14686996.2021.1916378

**Published:** 2021-05-28

**Authors:** Hisashi Ozaki, Ryo Hamai, Yukari Shiwaku, Susumu Sakai, Kaori Tsuchiya, Osamu Suzuki

**Affiliations:** a Division of Craniofacial Function Engineering, Tohoku University Graduate School of Dentistry, Sendai, Japan; b Department of Dentistry, Oral and Maxillofacial Surgery, Yamagata Prefectural Central Hospital, Yamagata, Japan; c Liaison Center for Innovative Dentistry, Tohoku University Graduate School of Dentistry, Sendai, Japan

**Keywords:** Autograft, autologous bone, octacalcium phosphate, complex, mechanical mixing, bone regeneration, intramembranous bone, dissolution, mineralization, 30 Bio-inspired and biomedical materials, 107 Glass and ceramic materials

## Abstract

This study examined the effect of a mixture of octacalcium phosphate (OCP) and autologous bone on bone regeneration in rat calvaria critical-sized defect (CSD). Mechanically mixed OCP and autologous bone granules (OCP+Auto), approximately 500 to 1000 μm in diameter, and each individual material were implanted in rat CSD for 8 weeks, and subjected to X-ray micro-computed tomography (micro-CT), histology, tartrate-resistant acid phosphatase (TRAP) staining, and histomorphometry for bone regeneration. Osteoblastic differentiation from mesenchymal stem cells (D1 cells) was examined in the presence of non-contacting materials by alkaline phosphatase (ALP) activity for 21 days. The material properties and medium composition before and after the incubation were determined by selected area electron diffraction (SAED) under transmission electron microscopy (TEM), Fourier transform infrared (FT-IR) spectroscopy, scanning electron microscopy (SEM), and chemical analysis. The results showed that while bone formation coupled with TRAP-positive osteoclastic resorption and cellular ALP activity were the highest in the Auto group, a positive effect per OCP weight or per autologous bone weight on ALP activity was found. Although the OCP structure was maintained even after the incubation (SAED), micro-deposits were grown on OCP surfaces (TEM). Fibrous tissue was also exposed on the autologous bone surfaces (SEM). Through FT-IR absorption, it was determined that bone mineral-like characteristics of the phosphate group increased in the OCP + Auto group. These findings were interpreted as a structural change from OCP to the apatitic phase, a conclusion supported by the medium degree of saturation changes. The results demonstrate the mutual chemical effect of mixing OCP with autologous bone as an active bone substitute material.

## Graphical abstract

**Figure:**
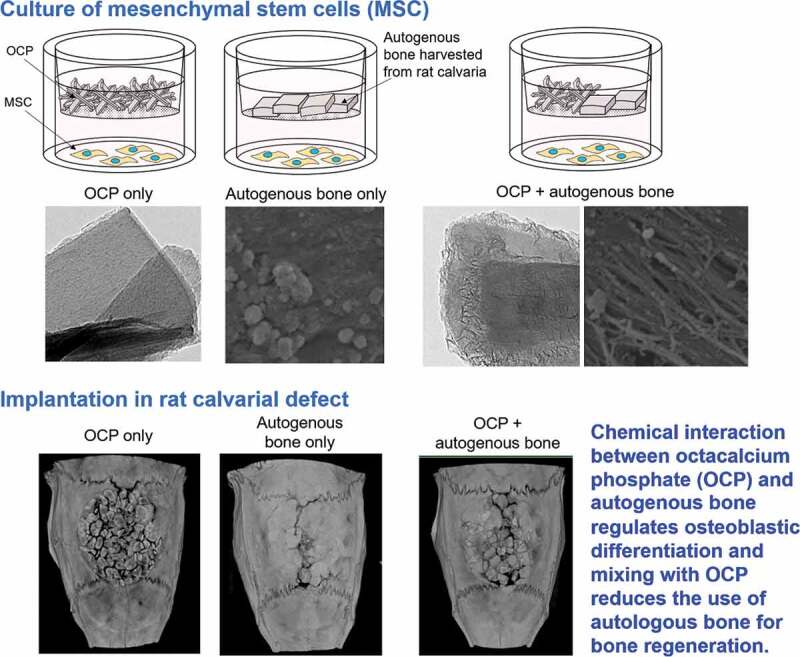

## 1. Introduction

The first choice for filling various bone defects is still autologous bone because of its superior osteoinductive and osteoconductive performance in bone regeneration [1]. This superior bone regenerative performance is a results of the fact that autologous bone contains osteoblastic cells, growth factors such as bone morphogenetic protein (BMP), matrix materials such as collagen, non-collagenous proteins, and inorganic calcium phosphate (CaP) apatite crystals [1,2]. However, due to the limitations of harvesting autologous bone and the possibility of complications, allografts [3], xenografts [4] and synthetic bone substitute materials [1] have been utilized. Synthetic materials mimicking the chemical composition of bone apatite have been developed, including hydroxyapatite (HA) [5,6], usually considered the prototype for bone apatite crystals, β-tricalcium phosphate (β-TCP) [7], biphasic HA/β-TCP [8] and non-stoichiometric bone-apatite-mimicking Ca-deficient HA (CDHA) [9], carbonate apatite [10], and these synthetic materials combined with polymeric materials, such as collagen [11] and poly-L-lactic acid (PLLA) [12]. These bone-apatite-mimicking materials have a direct bone-bonding property called osteoconductivity [13,14]. A great deal of effort has gone into reducing the amount of autologous bone that must be harvested by combining these synthetic materials with autologous bone.

The formation of bone apatite crystals, consisting of nano-sized CaP, has been studied and explained by a process in which the initial crystals are formed within membrane-bound matrix vesicles (MVs) secreted by osteoblasts [15–19]. After this, the crystals grow outside of the MVs by interacting with non-collagenous proteins, such as osteocalcin [20], and finally are deposited within collagen matrices synthesized by osteoblasts to form bone tissue [21]. The tissue fluid contains Ca^2+^ and inorganic phosphate (Pi) ions, which are the primary elemental ions for bone apatite crystals, and other ions such as magnesium, carbonate, and fluoride ions, the fluid of which provides a driving force for apatite crystal nucleation and growth [22]. Biological apatite crystals are formed by involving these ions in the structure, thereby becoming non-stoichiometric in composition, which in turn affects crystal’s chemical and physical properties, such as apparent solubility and crystal morphology [2]. It is known that the tissue fluid is supersaturated with respect to HA and almost saturated with respect to another CaP phase, octacalcium phosphate (OCP) [23,24]. During the bone remodeling process effected by the functions of osteoblasts and osteoclasts, bone apatite crystals can be chemically dissolved through an acidic attack yielded from osteoclasts [25]. Although the stability of bone apatite crystals in autologous bone may be susceptible to biodegradability in the synthetic materials if mixing at filling bone defects with them, it is not known whether the presence of such synthetic materials affects bone apatite chemical, physical, or biological properties in autologous bone. Thus, it is also unknown how the bone regenerative property of autologous bone is controlled and, conversely, how autologous bone strengthens or weakens the biological performance of the materials.

OCP is an acidic CaP material that includes 33% HPO_4_ per total phosphorus and contains many water molecules in its structure with a chemical formula Ca_8_H_2_(PO_4_)_6_ ·5H_2_O [26]. OCP has non-stoichiometric forms, such as Ca_16_H_4+X_(PO_4_)_12_(OH)_X_·(10-X)H_2_O [27], as well as HA materials and exhibits structural Ca deficiencies and labile forms regarding the existence of HPO_4_, which accompanies reversible structural reordering [26]. OCP has been suggested as a precursor to bone apatite crystals [20,28,29] although its existence in bone is still controversial [30]. OCP, which belongs to the triclinic system with the space group P1 [27], consists of apatite layers alternately stacked with hydrated layers and can be transformed to HA by utilizing the hydrated layers to hydrolyze and form new apatite crystals within the lattice [27,28]. It is considered that the solubility of OCP is higher than those of biodegradable β-TCP and the most thermodynamically stable HA under physiological pH [14]. The superior osteoconductivity of OCP was first found through its onlayed implantation onto mouse calvaria, while structural change to the HA phase took place progressively in situ on the calvaria in contrast to CDHA and stoichiometric HA [31]. The prominent osteoconductive performance of OCP has recently led to clinical application in some cases together with certain natural polymers [32,33]. Further studies have elucidated that the conversion to HA from OCP under physiological conditions involves a mechanism that induces osteoblastic cell differentiation [34,35], osteocyte differentiation [36] and osteoclast formation by OCP [37]; the latter property of osteoclastogenesis causes biodegradability in OCP [37]. It was found that when OCP is hydrolyzed to HA, the surrounding pH gradually decreases to some extent with the advancement of the hydrolysis [38]. A recent study found that a subtle pH decrease of OCP controls the hydrolysis rate of poly (lactic-co-glycolic acid) (PLGA) and affected the bone regeneration capacity of a PLGA-OCP composite [39]. This suggests that such an intrinsic chemical characteristic of OCP may affect not only synthetic material deterioration around OCP, but also the characteristics of biological materials such as bone apatite crystals in autologous bone if they are mixed into bone defects, thereby modulating their biological performance.

Based on previous studies and the hypothesis above, the present study was designed to investigate bone regeneration by implanting autologous bone harvested from rat calvaria mixed with OCP into rat calvaria critical-sized defects by analyzing the interaction from the viewpoint of chemical dissolution of the inorganic phases, crystallographic changes in OCP, and biological responses of osteoblastic cells and intramembranous bone tissues to autologous bone and OCP.

## 2. Materials and methods

### 2.1. Preparation of OCP granules

OCP was prepared using the wet synthesis method according to previous literature [31]. Briefly, OCP was precipitated by mixing a calcium acetate solution and an inorganic phosphate solution under a specific supersaturation condition with respect to OCP. The precipitate was separated from the solution and then washed with deionized water. The precipitate was dried at 105°C overnight. The dried precipitate was ground and subsequently passed through a standard test sieve to fabricate granules with diameters of 500–1000 μm. The granules were sterilized in a dry sterilizer at 120°C for 2 h.

### 2.2. Animal experiments

Twelve weeks old Wistar male rats (Japan SLC, Inc. Hamamatsu, Japan) were used for animal experiments to examine the bone formation behavior of the calvarial defects treated with OCP granules, autogenous bone granules, and a mixture of the two. All procedures for animal handling and treatment in this study were reviewed and approved by the Animal Research Committee of Tohoku University (approval number 2018DnA-036). The protocol conformed to all principles of laboratory animal care and national laws. Rats were anesthetized by inhalation of isoflurane, followed by the intraperitoneal injection of three types of mixed anesthetic agents (0.38 µg/g of medetomidine, 2.0 µg/g of midazolam, and 2.5 µg/g of butorphanol relative to the weight of the rat). The skin was sectioned along the bilateral line and the middle of the forehead, and the periosteum of the calvaria was ablated. A full-thickness standardized defect with a diameter of 9 mm was created using a trephine drill during the injection of saline, and bone fragments on the dura mater of the brain were collected. The created defect was located on the line sagittal suture between the lambdoid suture and coronal suture. The harvested calvarial bone (autogenous bone) was cut into granules of approximately 1 mm × 1 mm. 10.0 mg OCP granules (OCP group, n = 5), 42.7 mg autogenous bone granules (Auto group, n = 5), and a mixture of 5.0 mg OCP and 21.4 mg autogenous bone granules (OCP + Auto group, n = 5) were implanted into the defect. Defects without implantation were also created as a negative control (n = 5) using the same method as for the implantation of the granules. The ablated periosteum and skin were repositioned and sutured after the implantation of the granules or creation of the defect without implantation. At 2, 4, and 8 weeks of implantation, the calvarial tissue with and without implantation of the granules collected from the sacrificed rats were fixed with 10% formalin solution for 1 day.

### 2.3. Radiographic analysis

The fixed tissues were analyzed with a microfocus X-ray computed tomography system (micro-CT; Scan XmateE090, Comscantecno Co., Ltd., Kanagawa, Japan) at 90 kV and 100 μA. The micro-CT images were analyzed using CT image reconstruction operation software (coneCTexpress I, White Rabbit Co., Ltd., Tokyo, Japan) and 3-D image analysis software (Amira 6, Maxnet, Tokyo, Japan).

### 2.4. Histomorphometric analysis

The fixed tissues were decalcified in ethylenediaminetetraacetic acid solution at a pH of 7.1 for 4 weeks. The tissues were also dehydrated with a graded series of ethanol and then embedded in paraffin. The region of calvaria including the center of the defect was extracted and sectioned coronally with a thickness of 4–5  μm. The sections were stained with hematoxylin and eosin (H-E). Subsequently, the sections were observed using a slide scanner (NanoZoomer-SQ, Hamamatsu Photonics K. K., Shizuoka, Japan). The newly formed bone rate (n-bone%) was calculated as ({newly formed bone area [mm^2^]/original defect area [mm^2^]} × 100) by analyzing the microscope image of the section using software (Image J; National Institutes of Health, Bethesda, MD, USA).

### 2.5. Tartrate-resistant acid phosphatase staining

Tartrate-resistant acid phosphatase (TRAP) staining was performed to detect the osteoclast-like cells around autogenous bone and OCP. TRAP solution was prepared by mixing 40 mM sodium acetate, 50 mM sodium tartrate, naphthol AS‐MX phosphate, and N,N‐dimethyl formamide (all obtained from Sigma), and adjusted to pH 5.0. The sections were stained with Fast Red Violet LB (Sigma) dissolved in TRAP solution for 1 h at 37°C. Samples were counterstained with hematoxylin. The stained sections were observed using a slide scanner (NanoZoomer-SQ, Hamamatsu Photonics K. K., Shizuoka, Japan).

### 2.6. Cell culture experiments

Mouse bone marrow-derived mesenchymal stem cell (MSC) line D1 cells (ATCC, Rockville, MD, USA) were seeded on a 24-well plate at 2.0 × 10^4^ cells/well in 500 μL of osteogenic medium. The medium consisted of Dulbecco’s Modified Eagle’s Medium (FUJIFILM Wako Pure Chemical Co.) containing 10% fetal bovine serum (ThermoFisher Scientific, Waltham, US), 1% penicillin-streptomycin mixed solution (Nacalai Tesque, Inc., Kyoto, Japan), 50 μg/mL ascorbate 2-phosphate (Sigma-Aldrich, St. Louis, MO, USA), 10 mM β-glycerophosphate (Tokyo Chemical Industry Co., Ltd., Tokyo, Japan), and 100 nM dexamethasone (Sigma-Aldrich). Transwell inserts with 8.0 μm pores (FALCON® Cell Culture Insert, Corning Co.) were set into each well. Four milligrams of OCP granules, 4 mg of harvested calvarial bone granules from the rat (referred to as calvarial bone hereafter), and a mixture of 2 mg OCP and 2 mg harvested calvarial bone were placed on the bottom of the inters, after which 500 μL of osteogenic medium was added. The D1 cells with OCP (OCP group, n = 3), calvarial bone (Auto group, n = 3), and mixture (OCP + Auto group, n = 3) were cultured at 37°C in a humidified incubator with an atmosphere of 5% CO_2_ and 95% air. The D1 cells without OCP and calvarial bone were also incubated as a control group (n = 3). The osteogenic medium was changed every three days. At 7, 14, and 21 days, the incubated cells on the wells were washed with PBS and suspended in 250 μL Triton X-100 solution. Subsequently, the cells were crushed using a sonicator in an ice bath. The alkaline phosphatase (ALP) activities and DNA concentrations in the cell lysates were determined using LabAssay ALP® (FUJIFILM Wako Pure Chemical Co., Osaka, Japan) and a Quant-iT™ PicoGreen® dsDNA kit (Thermo Fisher Scientific), respectively. The value of ALP activity was normalized by the DNA concentration of the cells to compare the osteoblastic differentiation of the MSCs cultured with the granules. Furthermore, the normalized ALP activity per unit weight of OCP or calvarial bone was calculated. For the OCP + Auto group, the activity per weight values of OCP and calvarial bone were estimated by (1) and (2), respectively:

(1) $$ALP\;per\;weight\;of\;OCP=\frac{A_{OCP+Auto}-\left(\frac{A_{Auto}}{W_{Auto}}\right)\times 2}{W_{OCP\;in\;OCP+Auto}}$$

(2) $$ALP\;per\;weight\;of\;calvalial\;bone=\frac{A_{OCP+Auto}-\left(\frac{A_{OCP}}{W_{OCP}}\right)\times 2}{W_{Auto\;in\;OCP+Auto}}$$

where *A*_OCP+ Auto_, *A*_Auto,_ and *A*_OCP_ are the ALP activity for the OCP + Auto, Auto, and OCP groups, respectively. *W*_Auto_ and *W*_OCP_ are the weights of the granules incubated for the Auto and OCP groups, respectively. The terms (*A*_Auto_/*W*_Auto_) × 2 and (*A*_OCP_/*W*_OCP_) × 2 denote the activity per 2 mg of the calvarial bone and OCP granules, respectively. Thus, the numerators in Equations (1) and (2) correspond to the estimated ALP activity induced by OCP only and by calvarial bone only in the mixture, respectively. *W*_OCP in OCP+ Auto_ and *W*_Auto in OCP+ Auto_ are the weight of OCP and calvarial bone in the mixture, respectively.

The concentrations of Ca^2+^ and inorganic phosphate (Pi) ions in the supernatants of the collected medium were measured using the Calcium E test Wako and Phosphor C test Wako (FUJIFILM Wako Pure Chemical Co.), respectively. The pH values of the supernatants were also measured using a pH electrode (9618S-10D, HORIBA Ltd., Kyoto, Japan) at 37°C. The degree of supersaturation (DS) with respect to calcium phosphates was estimated based on the measurement results of ion concentration and pH at 37°C. The values of DS with respect to HA, OCP, and calcium hydrogen phosphate dihydrate (DCPD) were calculated using Equations (3)–(5), respectively:

(3) $$DS={\left(\frac{{\left[Ca^{2+}\right]}^{5}{\left[PO_{4}^{3-}\right]}^{3}\left[OH^{-}\right]}{K_{sp}}\right)}^{\frac{1}{9}}$$

(4) $$DS={\left(\frac{{\left[Ca^{2+}\right]}^{4}\left[H^{+}\right]{\left[PO_{4}^{3-}\right]}^{3}}{K_{sp}}\right)}^{\frac{1}{8}}$$

(5) $$DS={\left(\frac{\left[Ca^{2+}\right]\left[HPO_{4}^{2-}\right]}{K_{sp}}\right)}^{\frac{1}{2}}$$

where *K*_sp_ is the solubility product constant. The constants with respect to HA, OCP, and DCPD are 7.36 × 10^−60^ (mol·L^−1^)^9^ [40], 2.51 × 10^−49^ (mol·L^−1^)^8^ [41] and 2.77 × 10^−7^ (mol·L^−1^)^2^ [42], respectively. In these calculations, the presence of ion pairs (CaH_2_PO_4_^+^, CaHPO_4_^0^, MgHPO_4_^0^, CaHCO_3_^+^, and MgHCO_3_^+^) was considered to obtain the three mass balance values for Ca^2+^, Pi ion, and Mg^2+^. The ion strength was set at 0.15 M based on the background electrolyte of 150 mM Na^+^. DS values of 1.0, below 1.0, and above 1.0 indicate saturation, undersaturation, and supersaturation, respectively.

### 2.7. Characterization of OCP and harvested calvarial bone

The prepared granules of OCP and autogenous bone granules harvested from the rat calvarial bone were observed using SEM (JSM-6390LA, JEOL Ltd.) with an accelerating voltage of 10 kV.

The OCP granules, calvarial bone granules, and mixture were collected after incubation with D1 cells at 14 days. The lyophilization of the collected specimens was carried out after washing them with ultrapure water several times. The specimens were observed before and after incubation using a transmission electron microscope (TEM; JEM-2100F, JEOL Ltd., Tokyo, Japan) at an acceleration voltage of 100 kV. Crystallographic analysis of the incubated OCP and calvarial bone was also performed by selected area electron diffraction (SAED). The surface morphology of the incubated calvarial bones with and without OCP was observed using SEM (JSM-6390LA, JEOL Ltd.). Fourier transform infrared spectroscopy (FT-IR) spectra of the incubated specimens were measured using FT-IR spectroscopy (FT/IR-6300; JASCO Corporation, Tokyo, Japan) with the specimens diluted in KBr.

### 2.8. Statistical analysis

Results are expressed as mean ± standard deviation (SD). Tukey−Kramer multiple comparison analysis was carried out to evaluate statistical differences using data analysis software (Statcel2 software for Excel). A value of *p* < 0.05 was considered statistically significant.

## 3. Results

### 3.1. Microstructure of OCP and harvested calvarial bone granules

**Figure 1.**
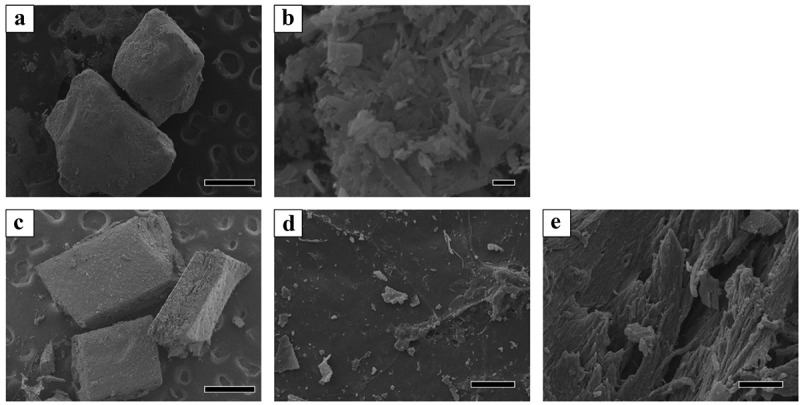
SEM images of OCP and harvested rat calvarial bone granules. Lower (a) and higher magnified image (b) of OCP granules. Bars in the lower and higher magnified image represent 500 and 1 μm, respectively. Overview (c), surface (d), and cross-section (e) images of the calvarial bone. Bars in the overview, surface, and cross-section images represent 500 and 100 μm, respectively

The microstructure of the prepared OCP granules and bone granules harvested from the rat calvaria (Auto) is displayed in the SEM images (Figure 1). The diameter of the observed OCP granules was greater than 500 μm (Figure 1(a)). In the OCP granules, the plate-like particles elongated toward the long axis were aggregated (Figure 1(b)). The calvarial bone granules had a cuboid shape with cross-sectional dimensions of 700–1000 μm and a thickness of approximately 300 μm (Figure 1(c)). Although the harvested bone surface of the periosteum or dura mater side seemed to be smooth (Figure 1(d)), a fibrous structure oriented in the direction orthogonal to the direction of the thickness was observed on the cross-section (Figure 1(e)).

### 3.2. Animal experiments

#### 3.2.1. Micro-CT analysis

**Figure 2.**
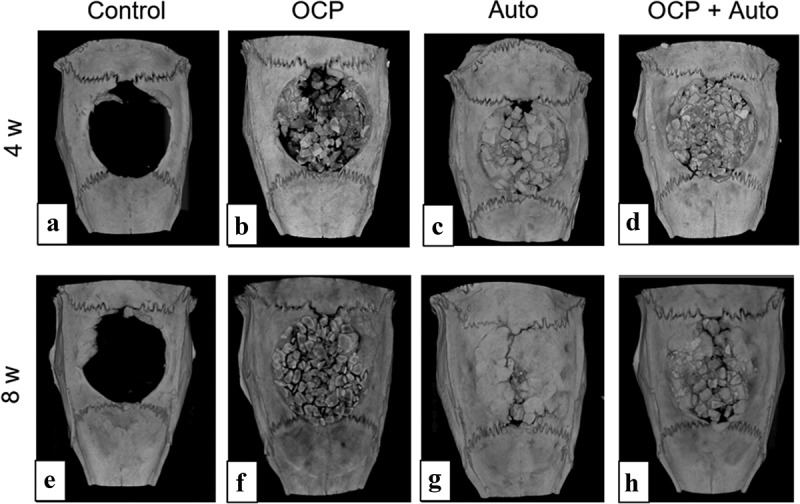
Micro-CT images of rat calvarial bone defects without treatment (a, e) and with implantation of OCP (b, f), autogenous bone (c, g), and mixture of OCP and autogenous bone (d, h) at 4 weeks (a–d) and 8 weeks (e–h)

The rat calvarial bone defects, including those of the untreated group (control group), the group treated with OCP and autogenous bone granules (referred to as autogenous bone hereafter) (Auto group), and the group treated with the mixture of OCP and autogenous bone (OCP + Auto group) were visualized by micro-CT at 4 and 8 weeks post-implantation (Figure 2). In the control group, radiopaque tissues were not observed in most areas of the bone defect at 4 weeks (Figure 2(a)), and the area of radiopacity was limited to the edge of the defect at 8 weeks (Figure 2(e)). The radiopacity in the Auto (Figure 2(c)) and OCP + Auto (Figure 2(d)) groups were higher than that in the OCP group (Figure 2(b)) at 4 weeks. In the Auto and OCP + Auto groups at 4 weeks, the radiopacity level tended to increase among the more radiopaque parts in a block shape. In OCP groups, the radiopacity seemed to increase at 8 weeks compared to that at 4 weeks, but was still lower than that of the Auto and OCP + Auto groups at 8 weeks. Most areas of the defect exhibited radiopacity similar to that of the intact bone area in the Auto group at 8 weeks, although such radiopacity areas were limited around the edge of the defect in the Auto + OCP group.

#### 3.2.2. Histological and histomorphometric analysis

**Figure 3.**
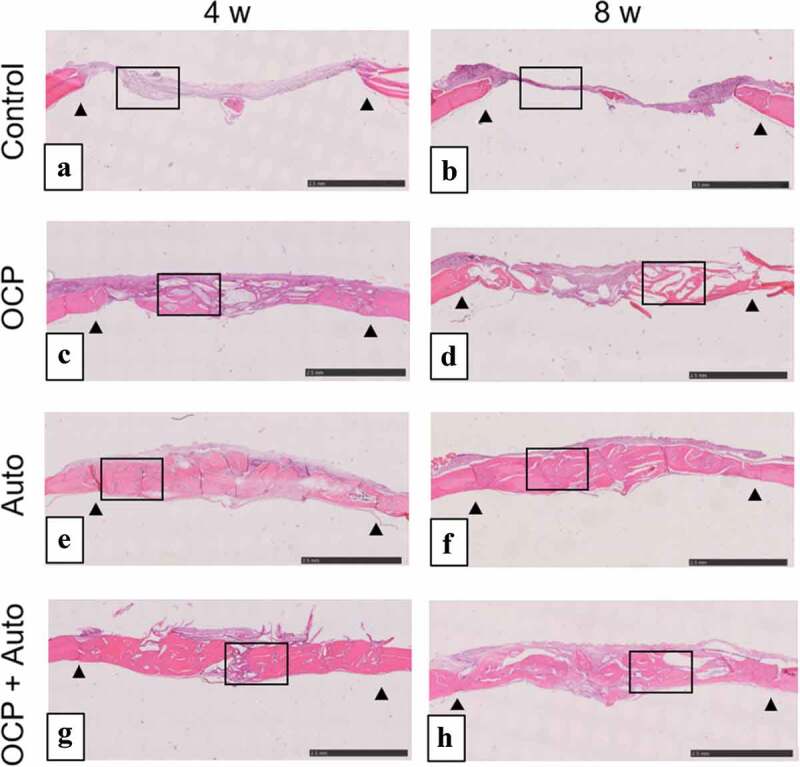
Lower magnified images of rat calvarial defect regions in the sections with haematoxylin-eosin staining at 4 weeks (a, c, e, g) and 8 weeks (b, d, f, h) of implantation of no materials (Control) (a, b), OCP (c, d), autogenous bone (e, f), and mixture of OCP and bone (g, h). Bars in the images represent 2.5 mm. Arrow heads indicate the edges of bone defects

**Figure 4.**
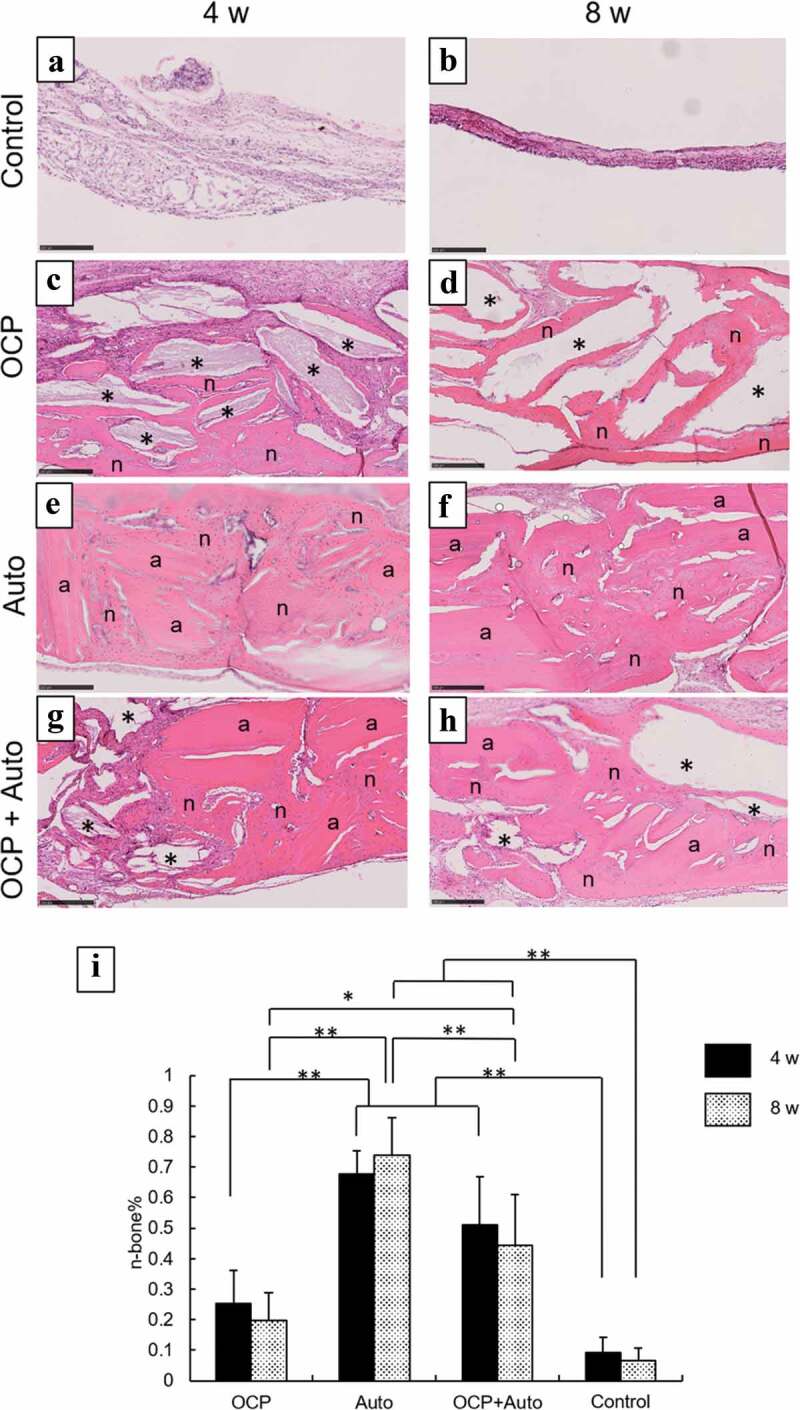
Higher magnified images of rat calvarial defect regions in the sections with haematoxylin-eosin staining at 4 weeks (a, c, e, g) and 8 weeks (b, d, f, h) of implantation of no materials (Control) (a, b), OCP (c, d), autogenous bone (e, f), and mixture of OCP and bone (g, h). Bars in the images represent 250 μm. Asterisks and ‘a’ indicate remaining OCP and autogenous bone; ‘n’ indicates newly formed bone, respectively. Histomorphometric analysis of newly formed bone in defects without and with implantation of OCP, autogenous bone (Auto), and mixture of OCP and Auto at 4 and 8 weeks (i) (*p < 0.05, **p < 0.01)

The behavior of new bone formation in the calvarial defects was also examined by histomorphometric analysis using H-E staining of the sections (Figures 3 and 4). The thin connective tissue was filled at 4 weeks (Figures 3(a) and 4(a)) and the limited formation of new bone was observed close to the edges of the defect at 8 weeks (Figures 3(b) and 4(b)) in control groups. In the lower magnified views, newly formed bone tissues were observed in the OCP, Auto, and OCP + Auto groups at 4 weeks (Figure 3(c,e,g)). The new bone tissue bridged the defect at 4 and 8 weeks in the Auto group (Figure 3(f)), although the few connective tissue seemed to remain at the center of the defect in the OCP and OCP + Auto groups at 8 weeks (Figure 3(d,h)).

In the more heavily magnified views, the remaining autogenous bone were observed to exhibit an oriented lamellar structure in the Auto and OCP + Auto groups at 4 weeks, similar to the intact bone structure (Figure 4(e,g)). The OCP granules stained by hematoxylin also remained in the OCP and OCP + Auto groups (Figure 4(c,g)). The newly formed bone directly contacted the remaining OCP and autogenous bone at 4 weeks, although the thickness of new bone on the autogenous bones seemed to be higher than that on OCP. The boundaries between the autogenous bone and new bone tissue tended to be indistinct in the Auto and OCP + Auto groups at 8 weeks compared to those at 4 weeks (Figure 4(f,h)). In the OCP groups, the number of granules surrounded by the newly formed bone tended to be larger at 8 weeks than that at 4 weeks (Figure 4(d)).

Histomorphometric analysis of the sections was also performed to quantify the rate of the newly formed bone tissue (n-bone%) in the defect with and without treatment at 4 and 8 weeks (Figure 4(i)). The n-bone% values for OCP, Auto, and OCP + Auto groups were higher than that for the control group at 4 weeks, while the Auto group showed higher n-bone% compared to the other groups, and the rate for the OCP + Auto group ranked between the Auto and OCP groups. There was a significant difference between the control and Auto groups as well as between control and the OCP + Auto group. A significant difference was also observed between the OCP and Auto groups as well as between the OCP and OCP + Auto groups, although there was no significant difference between the Auto and OCP + Auto groups.

The magnitude relation of the n-bone% values among these groups at 8 weeks was the same as that of the values at 4 weeks. The n-bone% for the Auto group at 8 weeks tended to increase compared to that at 4 weeks, although these values for the OCP, Auto + OCP, and control groups slightly decreased at 8 weeks compared to those at 4 weeks. The n-bone% for the Auto group was significantly higher than that for the control, OCP, and OCP + Auto groups at 8 weeks. A significant difference was also observed between the OCP and Auto groups as well as the OCP + Auto group. However, there was no significant difference between the control and OCP groups.

#### 3.2.3. TRAP staining

**Figure 5.**
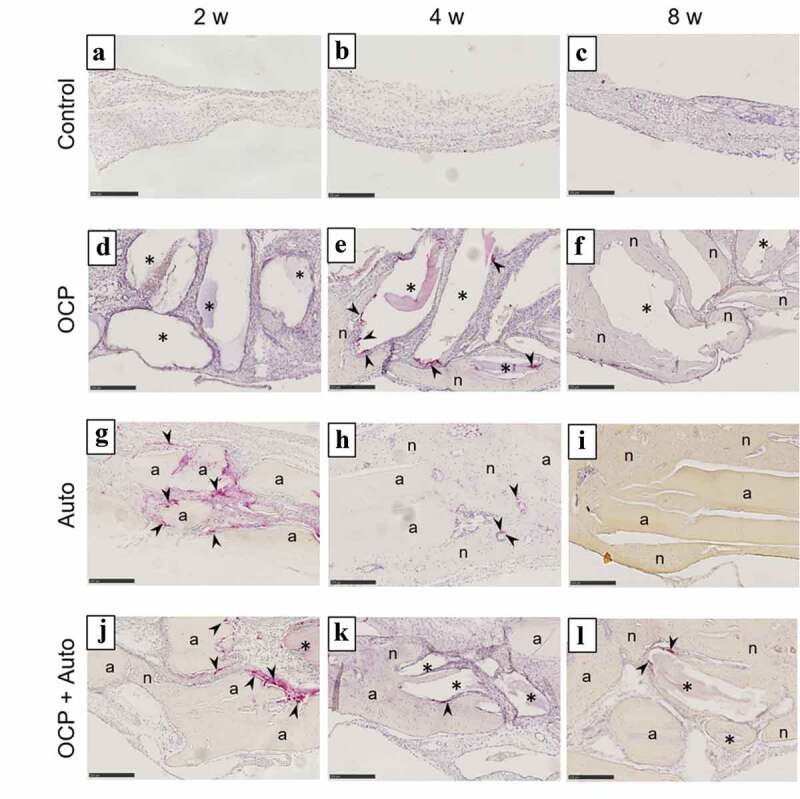
Images of rat calvarial defect regions in the sections with TRAP staining at 2 weeks (a, d, g, j), 4 weeks (b, e, h, k), and 8 weeks (c, f, i, l) of implantation of no materials (Control) (a–c), OCP (d–f), autogenous bone (g–i), and mixture of OCP and bone (j–l). Bars in the images represent 250 μm. Arrow heads indicate TRAP-positive cells; asterisks and ‘a’ indicate remaining OCP and autogenous bone; ‘n’ indicates newly formed bone, respectively

To examine the induction behavior of osteoclast-like cells in the bone defects with and without implantations, TRAP staining of the sections was carried out (Figure 5). There were no TRAP-positive multinucleated cells in the control group until 8 weeks had elapsed (Figure 5(a–c)). At 2 weeks, TRAP-positive multinucleated cells appeared around the remaining Auto and OCP granules in the Auto (Figure 5(g)) and OCP + Auto groups (Figure 5(j)). However, positive cells were not detected in the OCP group at 2 weeks (Figure 5(d)). Positive cells surrounded by the newly formed bone and attached to the remaining OCP were observed in the Auto (Figure 5(h)) and OCP + Auto groups (Figure 5(k)), respectively, at 4 weeks. In the OCP group, the number of positive cells rapidly increased around the OCP granules at 4 weeks (Figure 5(e)). In contrast, the number of positive cells decreased in the Auto and OCP + Auto groups at 4 weeks compared to those at 2 weeks. Although positive cells were not observed in the OCP (Figure 5(f)) and Auto groups (Figure 5(i)) at 8 weeks, the presence of positive cells was detected in the remaining OCP and OCP + Auto groups (Figure 5(l)).

The number of the sections in which positive cells were detected was counted in the OCP, Auto, OCP + Auto, and control groups at each period of post-implantation (Table 1). In the control group, there was no TRAP-stained section between 2 to 8 weeks. Two TRAP-stained sections were observed in the Auto and OCP + Auto groups at 2 weeks. There was no such section in the OCP group at 2 weeks. In contrast, all sections were stained with TRAP in the OCP group at 4 weeks. The Auto and OCP + Auto groups showed lower numbers of TRAP-stained sections compared to that of the OCP group at 4 weeks. The stained sections did not appear in the OCP and Auto groups at 8 weeks. The OCP + Auto group alone showed one stained section at 8 weeks.

**Table 1.** Number of sections detecting TRAP-positive cells in bone defect with and without the implantation of OCP, autogenous bone (Auto), and mixture of the two

Samples	Implantation periods		
	2 w	4 w	8 w
OCP	0/5	5/5	0/5
Auto	2/5	1/5	0/5
OCP + Auto	2/5	2/5	1/5
Control	0/5	0/5	0/5

### 3.3. Cell culture experiments

#### 3.3.1. ALP activity of MSC incubated with OCP and harvested calvarial bones

**Figure 6.**
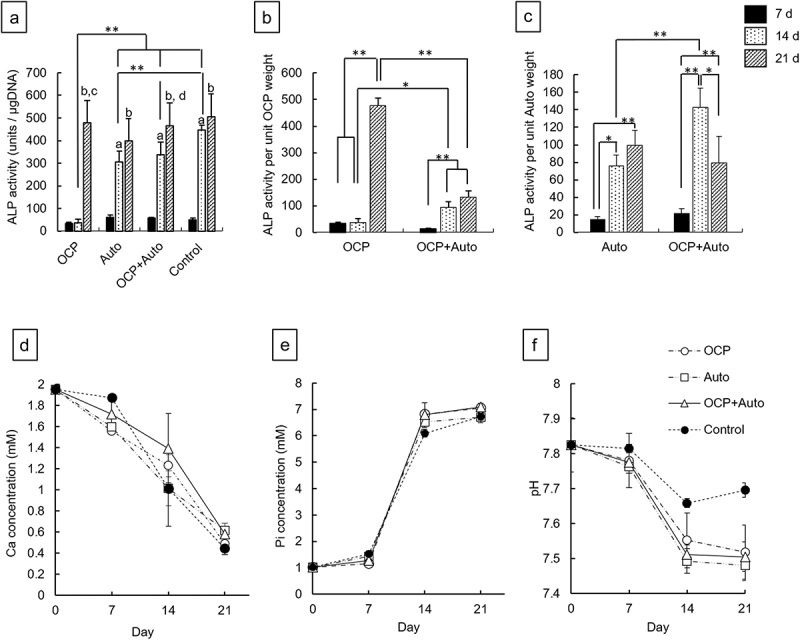
ALP activity of D1 cells without (control) and with incubation of OCP, harvested calvarial bone (Auto) and mixture of OCP and Auto at 7, 14, and 21 days (a). ALP activity per unit weight of OCP (b) and harvested calvarial bone granules (c) after incubation with OCP, Auto and mixture of them at 7, 14, and 21 days. (*p < 0.05, **p < 0.01, ^a^ significant difference (*p* < 0.01) between 7 and 14 days, ^b^ significant difference (*p* < 0.01) between 7 and 21 days. ^c^ significant difference (*p* < 0.01) between 14 and 21 days in OCP group, ^d^ significant difference (*p* < 0.05) between 14 and 21 days in OCP + Auto group). Change in Ca^2+^ (d), Pi ion (e) concentration and pH (f) in supernatants of osteogenic medium after the incubation with D1 cells

Osteoblastic differentiation induced by the presence of OCP, harvested calvarial bones (Auto), and OCP + Auto was examined by determining the ALP activity of MSCs in vitro (Figure 6(a)). The ALP activities for the Auto, OCP + Auto, and control groups increased significantly over 7 to 14 days. The activity in the OCP group tended to be maintained until 14 days had elapsed. The values of ALP activity for the Auto, OCP + Auto, and control groups were significantly higher than that for the OCP group at 14 days. The activities for the OCP + Auto group slightly increased compared to that of the Auto group, although a significant difference was not observed. Increasing ALP activity was observed in all groups over 14 to 21 days, while significant differences between 14 and 21 days were observed in the OCP and OCP + Auto groups. At 21 days, the activity values for the OCP and OCP + Auto groups tended to be higher than that for the Auto group. However, no significant differences were observed among the groups.

To determine the effects of the mixing of OCP and harvested calvarial bone on the osteoblastic differentiation of MSCs, the ALP activity per unit weight of OCP or harvested calvarial bone granules was estimated (Figure 6(b,c)). The ALP activity per unit weight of OCP in the OCP groups tended to be maintained over 7 to 14 days (Figure 6(b)). In contrast, the activity per unit OCP weight for the OCP + Auto group significantly increased at 14 days compared to that at 7 days. The estimated activity for the OCP + Auto group was significantly higher than that for the OCP group at 14 days, although there was no significant difference at 7 days. The activity per unit OCP weight significantly increased over 14 to 21 days in the OCP group as well as the OCP + Auto group. At 21 days, however, the activity per OCP weight for the OCP group was significantly higher than that for the OCP + Auto group. The activity per unit weight of calvarial bone for the Auto group increased with incubation time (Figure 6(c)). In contrast, the activity increased over 7 to 14 days and then decreased at 21 days in the OCP + Auto group. The estimated activity tended to be higher in the OCP + Auto group than in the OCP group at 7 and 14 days. A significant difference was observed only at 14 days.

#### 3.3.2. Change in ion concentration in culture medium

The concentration of ions in the medium after the cultures of MSCs in the OCP, Auto groups, and OCP + Auto groups was determined (Figure 6(d–f)). The Ca^2+^ concentration decreased with incubation time in the media for all groups (Figure 6(d)). The concentration of Ca^2+^ tended to be higher in the media incubated with OCP + Auto compared to OCP and Auto at 7 and 14 days. The concentration in the medium with incubation of OCP was also higher than that with Auto at 14 days. At 21 days, the concentrations of the OCP + Auto and Auto groups were slightly higher than that of the OCP group.

The Pi ion concentration for all groups increased slightly over 0 to 7 days (Figure 6(e)). The Pi in the medium with Auto was higher than that of OCP + Auto and OCP at 7 days. The ion concentration sharply increased over 7 to 14 days, and then a gradual increment of Pi was observed over 14 to 21 days. At 14 and 21 days, Pi concentrations in the OCP and OCP + Auto groups tended to be higher than those for the Auto group. The pH in all media decreased with incubation periods from 7.8 to around 7.5 (Figure 6(f)). The pH of the Auto group was slightly lower than that of the OCP and OCP + Auto groups at each incubation period.

Based on the results of ion concentrations and pH measurements, the values of DS with respect to calcium phosphates were calculated (Table 2). The DS with respect to the HA and OCP phases were on the orders of 10^13^–10^11^ and 10^5^–10^3^, respectively, in all media, which indicates that these media were supersaturated with respect to the HA and OCP phases. In contrast, the DS with respect to DCPD phase was on the order of 10^0^ or 10^–1^ regardless of the incubated materials and incubation period, which suggests that the media were saturated or undersaturated with respect to DCPD, respectively.

**Table 2.** Degree of supersaturation with respect to calcium phosphates in the cultures of D1 cells with the incubation of OCP and harvested calvarial bone (Auto) and mixture of the two

Supernatants	Periods (days)	DS at 37°C		
		HA	OCP	DCPD
Osteogenic medium	0	3.70 × 10^13^	1.64 × 10^4^	6.89 × 10^−1^
OCP	7	1.30 × 10^13^	8.07 × 10^3^	6.24 × 10^−1^
	14	3.65 × 10^13^	9.87 × 10^4^	2.34 × 10^0^
	21	3.08 × 10^11^	2.41 × 10^3^	9.70 × 10^−1^
Auto	7	2.25 × 10^13^	1.51 × 10^4^	7.89 × 10^−1^
	14	6.97 × 10^12^	2.99 × 10^4^	1.84 × 10^0^
	21	5.56 × 10^11^	4.16 × 10^3^	1.15 × 10^0^
OCP + Auto	7	2.73 × 10^13^	1.55 × 10^4^	7.52 × 10^−1^
	14	4.44 × 10^13^	1.27 × 10^5^	2.60 × 10^0^
	21	7.20 × 10^11^	4.78 × 10^3^	1.15 × 10^0^

The DS with respect to the HA and OCP phases for the Auto group decreased with incubation periods, although the DS for OCP and OCP + Auto groups decreased over 0 to 7 days and then slightly increased over 7 to 14 days. The DS with respect to HA was higher in the OCP + Auto group than in the other groups and the DS for the Auto group ranked between the OCP + Auto and OCP groups at each incubation period. The change in DS with respect to OCP displayed a tendency similar to that of the DS with respect to HA.

#### 3.3.3. Analysis of the incubated OCP and harvested bone granules

**Figure 7.**
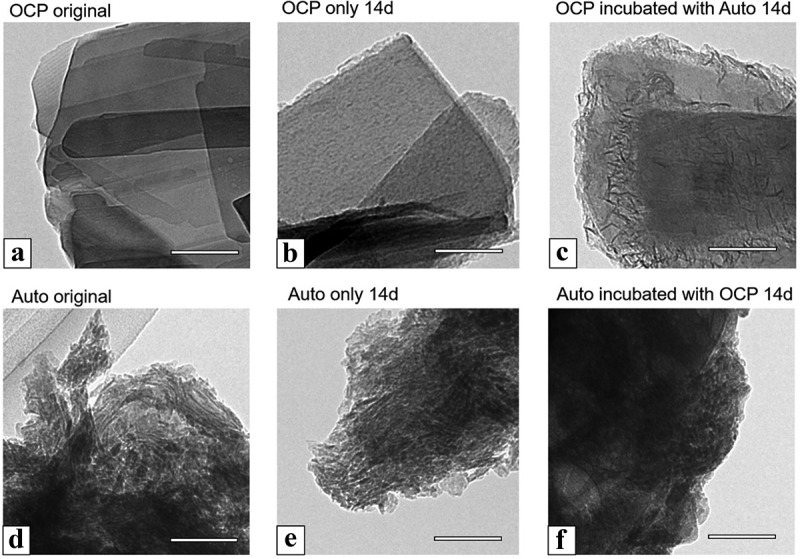
TEM images of OCP (a–c), harvested calvarial bone (Auto) (d–f) before (original) (a, d) and after incubation (b, c, e, f) in the osteogenic mediums for culture of D1 cells at 14 days. OCP incubated with Auto (c) and Auto incubated with OCP (f) in the medium. Bars in the images represent 100 nm

The OCP crystals and the harvested calvarial bones (Auto) before and after incubation with MSCs at 14 days were observed using TEM (Figure 7). In the bright-field image of the original OCP, as well as in the SEM image of OCP, a plate-like particle with a smooth surface was observed (Figure 7(a)). The plate-like shape was maintained after the incubation of OCP in the absence (Figure 7(b)) and presence (Figure 7(c)) of Auto at 14 days. Small depositions were observed on the edge of the plate-like particles incubated without Auto. After incubation with Auto, a larger amount of acicular deposition was formed on the edge of the plate-like particles of OCP. However, the nano-acicular particles were aggregated in the image of the original Auto (Figure 7(d)). The shape of each particle was also clearly observed in the image of Auto after incubation without OCP at 14 days (Figure 7(e)). In contrast, the shape of the particles in Auto tended to become unclear after incubation with OCP (Figure 7(f)).

**Figure 8.**
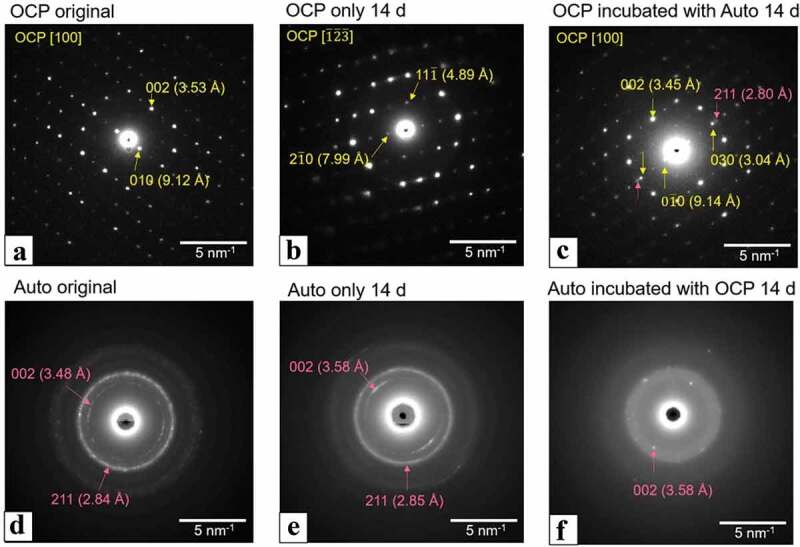
SAED patterns of OCP (a–c), harvested calvarial bone (Auto) (d–f) before (original) (a, d) and after incubation (b, c, e, f) in the osteogenic mediums with culture of D1 cells at 14 days. OCP incubated with Auto (c) and Auto incubated with OCP (f) in the medium. Bars in the images represent 5 nm^–1^. SAED patterns of original OCP (a), OCP incubated without (b) and with Auto (c) indicate the zone axis of [100], [123], and [100] OCP, respectively

The SAED images of OCP and Auto were obtained to analyze their crystal structure (Figure 8). The SAED pattern of the original OCP showed diffraction spots corresponding to 010 and 002 of OCP, which indicates that the orientation of the unit cell of OCP faced the same direction in the plate (Figure 8(a)). The SAED of Auto displayed the diffraction rings attributed to 211 and 002 of HA, which corresponds to the observation that the nano HA crystals randomly aggregated in Auto. The spots corresponding to the OCP structure were also detected after incubation with (Figure 8(c)) and without Auto (Figure 8(b)). However, a part of the ring with weak intensity was observed in the patterns of OCP incubated with Auto (Figure 8(c)), which was attributed to 211 of HA. The diffraction rings corresponding to HA were also observed in the SAED pattern of Auto incubated without OCP (Figure 8(e)), although the diffraction rings tended to be unclear after incubation in the presence of OCP (Figure 8(f)). These results suggest that the combination of OCP and Auto could promote the formation of *de novo* HA crystals on the plate-like OCP crystals and decrease the crystallinity of HA in Auto after incubation with MSCs.

**Figure 9.**
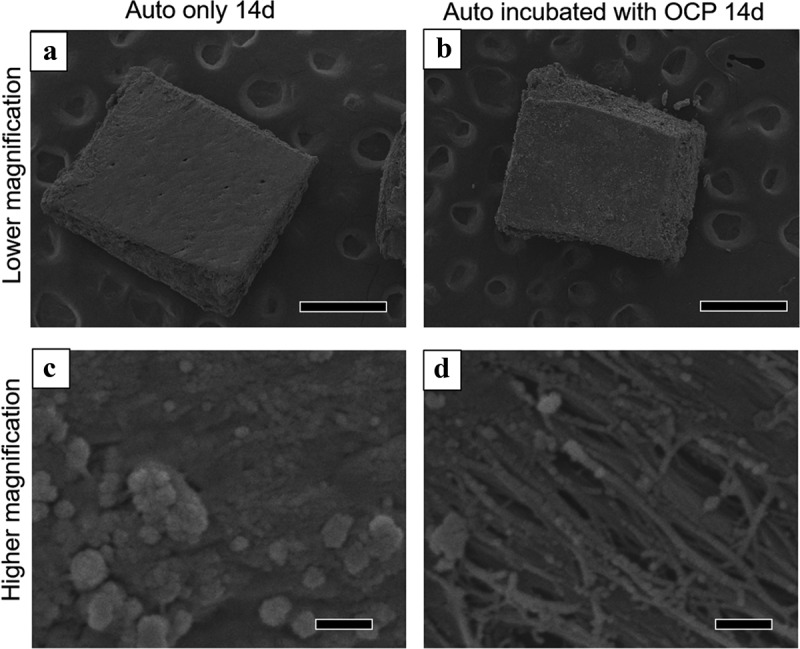
Lower (a, b) and higher (c, d) magnified SEM images of harvested calvarial bones (Auto) after incubation without (a, c) and with OCP (b, d) in the osteogenic medium at 14 days. Bars in the lower and higher magnified images represent 500 and 1 μm, respectively

The surface of Auto was also observed using SEM after incubation in the absence and presence of OCP (Figure 9). The lower magnified images show that Auto maintained a block-like shape after incubation, regardless of the presence of OCP at 14 days (Figure 9(a,b)). At higher magnification, spherical depositions with an approximate diameter of less than 1 μm were observed on the surface of Auto incubated without OCP (Figure 9(c)). The fibrous structure, which seemed to be collagen, appeared and the smaller particles were deposited on the fibers of Auto incubated with OCP (Figure 9(d)).

**Figure 10.**
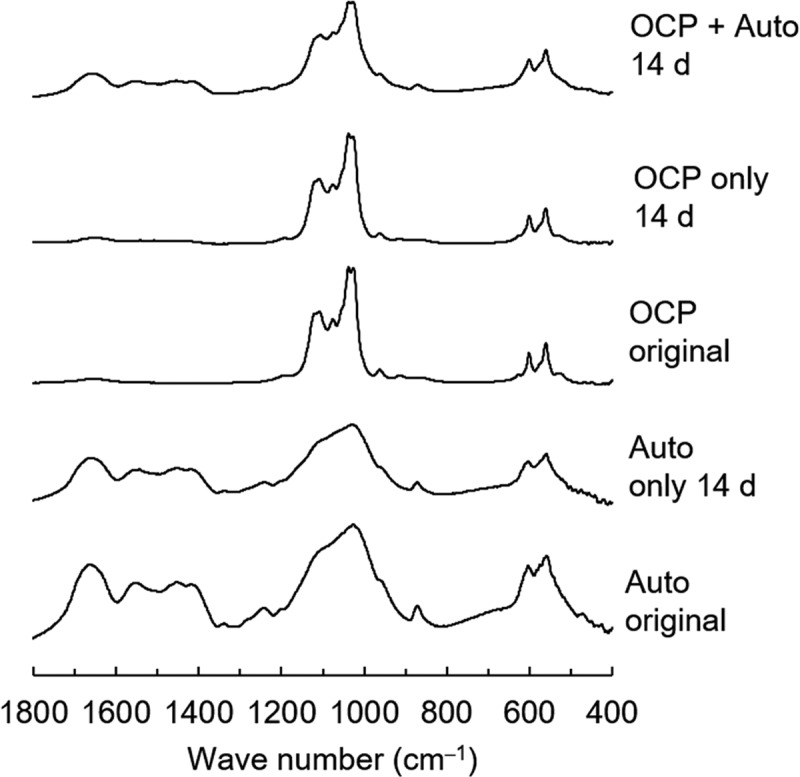
FT-IR spectra of OCP and harvested calvarial bone (Auto) before and after incubation in osteogenic medium as well as incubated mixture of OCP and Auto at 14 days

Furthermore, OCP, Auto, and the mixture of OCP + Auto were analyzed using FT-IR before and after incubation with MSCs (Figure 10). The peaks corresponding to ν_3_ and ν_1_ PO_4_ of apatite were detected at 1026 and 960 cm^–1^, respectively, in the FT-IR spectra of Auto before (original) and after incubation. Peaks attributed to amide I and ν_1_ CO_3_ were also observed at 1653 and 1560 cm^–1^, respectively, in both Auto spectra. The peaks in the split were detected at 1036 and 1024 cm^–1^ in the spectrum of the original OCP, which corresponded to ν_3_ PO_4_ of OCP. The spectrum of the original OCP also displayed a peak attributed to ν_3_ HPO_4_ and ν_3_ PO_4_ at 1076 cm^–1^. The split tended to become unclear in the spectra of OCP only after incubation. In the spectrum of the incubated OCP + Auto, the split including the peaks was completely unclear and the intensity of the peak at 1076 cm^–1^ decreased. Furthermore, the intensity of peaks attributed to ν_1_ CO_3_ tended to be lower in the spectrum of incubated OCP + Auto compared to Auto only after incubation.

## 4. Discussion

This study found that a mechanical mixture of OCP and autologous bone controls the capacity for new bone formation in each material. The dissolution and reprecipitation mechanism of OCP under the mixing condition was shown to enhance the dissolution of apatite crystals of autologous bone, resulting in increased osteoblastic cellular activity. OCP is a material that exhibits a higher osteoconductivity than HA or biodegradable β-TCP materials [31,35,43–45] while autologous bone is recognized to show the highest bone regenerative capacity among bone substitute materials [1,46–49]. The present study suggests that the chemical interaction between the inorganic phase OCP and bone apatite crystals plays a major role in activating the osteoconductive properties of each material.

The OCP used had an irregular shape, while autologous bone showed a block-like shape with a relatively smooth flat surface as well as a fracture surface (Figure 1). Both materials were prepared by mechanical grindings, but these shapes seem to reflect the intrinsic mechanical properties of these materials. Autologous bone is known to be composed of collagen and bone mineral and therefore could exhibit viscoelasticity [50]. Mechanically mixed artificial bone substitutes and autologous bone have been clinically applied to fill in bone defects [51–54]. Although OCP is homogeneously dispersed in vehicles such as natural polymer materials, such as collagen and gelatin [55,56], to improve handling in some cases, a mixture of OCP and autogenous bone was implanted in the defects without any vehicle in this study. Thus, this study enabled us to estimate the direct bone tissue response without using vehicles. Indeed, micro-CT observations revealed that the granules were sufficiently dispersed within the defects for comparative estimation as bone substitute materials (Figure 2). The radiopacity of the granules seemed to increase with the implantation period in the OCP, OCP + Auto, and Auto groups, ordered from least to most radiopacity, from 4 weeks over 8 weeks, suggesting that these materials have qualitatively distinct bone-forming capacities.

Histological sections confirmed that autologous bone in the Auto groups was surrounded by a large amount of new bone in 4 weeks (Figure 3) and integrated with the new bone, and the interface between autologous bone and newly formed bone became indistinguishable by 8 weeks (Figure 4), suggesting that some autologous bone is replaced with new bone through biodegradation despite using cortical bone, as previously reported [57]. It has been understood that while cancellous bone tends to not only show prominent bone regenerative capacity but also is replaced with new bone [52], the replaceability of cortical bone with newly formed bone is not sufficiently high compared to that of cancellous bone [48]; cancellous bone is generally selected clinically for autografts [52]. However, due to limitations on the availability of cancellous bone, cortical bone from the calvaria was chosen in the present animal model and its use demonstrated biocompatibility with new bone formation. Osteoconductivity was highest in the Auto group, followed by the OCP + Auto and OCP groups. The histological qualitative estimation was quantitatively substantiated by histomorphometric analysis (Figure 4(i)). A higher osteoconductivity of OCP was reported in some previous studies in which OCP acted as a starting locus of bone deposition [31] and enhanced bone formation more than HA or β-TCP [31,35,43–45]. By mixing OCP with autologous bone, active bone formation may orthotopically occur around OCP beyond the single use of OCP.

It is important to learn how autologous bone and OCP are resorbed and replaced with newly formed bone because both materials have the potential to be phagocytosed by osteoclasts or multinuclear TRAP-positive osteoclast-like cells [37,44,58,59], which should affect osteoblast cellular activity and bone formation via cellular coupling [60–67]. This is because autologous bone contains osteocytes, which are capable of producing the osteoclast-inducing soluble factor RANKL [68–70] while OCP is capable of increasing RANKL expression of osteoblasts [37], both of which could enhance osteoclast formation. In fact, TRAP-positive osteoclast-like cells appeared around both materials (Figure 5). The staining revealed clear differences between autologous bone and OCP: TRAP-positive cells accumulated in 2 weeks but rapidly disappeared in 4 weeks around autologous bone. In contrast, the positive cells were present even at 4 weeks around the OCP (Figure 5). From a chemical perspective, OCP cannot dissolve simply in physiological Tris-HCl buffer solutions by attaining an equilibrium state, as if the buffer solution contains a physiological concentration of Ca^2+^ and Pi ions, then it becomes slightly supersaturated with respect to OCP, and if the buffer does not contain these ions, then it becomes slightly undersaturated with respect to OCP [71]. The buffer maintains its own supersaturation level, and the driving force to convert to HA from OCP can be maintained; therefore, the transformation advances as observed in vivo implantation of OCP [31,43,72,73]. Such thermodynamically metastable characteristics of OCP are a cause of in vitro enhancement of osteoclast formation [37], in vivo recruitment of bone marrow macrophages [74], and the induction of TRAP-positive osteoclast-like cell accumulation in the prolonged implantation periods in the present study in rat calvaria (Figure 5 and Table 1). The relatively long-term appearance of TRAP-positive cells around OCP has also been reported in various animal bone defect models [44,45,58,59]. The present results suggest that mixing OCP with autologous bone may have an impact on bone formation, resorption of autologous bone, and degradation of OCP itself through a possible interaction between OCP and autologous bone.

To determine whether mixing OCP with autologous bone has an additional effect from OCP to autologous bone or a bidirectional impact between these two materials on osteoblastic cellular activity, ALP activity per unit weight of OCP or autologous bone was analyzed (Figure 6). Because the cell culture was carried out by placing OCP, OCP + Auto, and Auto groups apart from MSCs (D1) using a Transwell plate in the present study, the ALP activity detected could be primarily due to the effect of ionic dissolution from OCP or bone apatite crystals under physiological medium conditions [75]. Although the ALP activity change by Auto and OCP + Auto groups follows generally that of the tendency by control group (cells only) over the incubation period, the ALP activity by OCP caught up the tendency that by the control at 21 days (Figure 6(a)). The analysis of ALP activity per OCP weight (Figure 6(b)) showed that OCP contributed to increased ALP activity in OCP + Auto at 14 days (with a significant difference) and maximizing it at 21 days (without a significant difference). In contrast, the ALP activity per autologous bone weight (Figure 6(c)) was maximized at 14 days (with a significant difference) but decreased at 21 days compared to the result at 14 days. These results confirm that the bidirectional effect on the osteoblastic differentiation of D1 cells between these two materials is involved in the mixing and suggest that OCP within the OCP + Auto group continuously stimulates D1 cell activity throughout the culture period up to 21 days. The present in vitro model used a mixing ratio of 2 mg OCP to 2 mg autologous bone (one to one), while the in vivo model used a mixing ratio of 5 mg OCP to approximately 21 mg (approximately one to four) due to the limitation of the implantation calvaria defect volume condition and the recovery of autologous bone from the calvaria. Because of the difference in the mixing ratio between in vitro and in vivo models and excess volume of OCP used for in vitro study, which is derived mainly from the bulk density of OCP, it is suggested that the bone formation capability of the OCP + Auto group in vivo may be influenced by the mixing ratio.

In order to analyze the effect of ionic dissolution and possible CaP crystal deposition, the driving force of the medium after the D1 cell incubation was estimated by calculating the DS numerical values from the inorganic ion composition (Table 2). These results suggest that the dissolution of OCP affected that of autologous bone apatite crystals. While the DS of the medium by the Auto group with respect to HA and OCP decreased with incubation time, DS of that by OCP was higher at 14 days than at 7 days and 21 days, and the latter tendency was reflected in the OCP + Auto group. Because the DS of the medium by OCP + Auto with respect to OCP was maintained at higher values compared to the other two groups, it is conceivable that OCP affected the dissolution not only in autologous bone but also in OCP itself in the co-presence of autologous bone.

Spectroscopic, morphological, and crystallographic analyses of the materials verified further evidence regarding mixing OCP with autologous bone, in which the bidirectional effect impact was demonstrated (Figures 7 and 9). Although the SAED pattern of OCP detected the corresponding OCP phase (Figure 8), TEM observation showed new nanomaterial-like deposition, which could exhibit the weak diffraction of HA in SAED (Figure 8(c)) around the original OCP crystals if autologous bone is co-present (Figure 7). This strongly suggests an increase in the DS of the solution around the OCP crystals and the potential for new deposit nucleation [23,24,76–80]. SEM observation identified exposure of collagen-like fiber morphology on autologous bone. This possible bone surface (crystal) dissolution could be induced by the physicochemical environment induced by OCP that induces the dissolution and decrease of pH around OCP during transformation from OCP to HA [38]. FT-IR spectroscopic analysis also found a phosphate ion spectral change around 1000 to 1100 cm^−1^, which corresponded to the progress of OCP hydrolysis and formation of the apatitic phase, as previously reported by similar studies employing spectroscopy and X-ray diffraction analysis [43,73], indicating that the co-presence of autologous bone also affected the structural properties of OCP as well as the chemical dissolution properties (Table 2). Similar interactions between HA and DCPD or HA and OCP have been considered from the perspective of mineralization analysis to understand the CaP crystal dissolution-reprecipitation mechanism [81].

The interaction between autologous bone and OCP may involve some mechanisms controlling bone regeneration in the defects. The observed in vitro dissolution of autologous bone in the presence of OCP suggests possible increment of osteoblastic cellular activity in vivo through releasing some of growth factors, such as BMP, embedded in bone matrices [82] via the bone surface dissolution probably enhanced by the presence of OCP. Another interesting property of OCP reported so far is the capacity adsorbing serum derived proteins, such as α2HS-glycoporteins [83], apolipoprotein E (Apo E) and complement 3 (C3) [84] on the crystal surface and also within the space between the crystals in OCP granules. Apo E and C3 have been reported to have functions in osteoblast differentiation [85] and in osteoclastic cells recruitment [86], respectively, suggesting that OCP has a positive role not only for host bone-related cells but also for osteoblastic and osteoclastic cells associated with autologous bone through these adsorbed proteins. Other studies have shown that soluble amorphous calcium phosphate (ACP) [87], reactive calcium phosphate cements [88] and mineralized HA crystals together with magnesium oxide (MgO) [89] display significant effects on increasing osteogenic cellular activities. Although there is no direct comparative evidence on the performance from these materials to the present OCP, calcium phosphate materials, including OCP, may commonly stimulate the osteogenic cells through their chemical properties. The overall results of the present study provide a theoretical rationale to support the proposition that mixing OCP with autologous bone induces a bidirectional impact in each material and could be used as an active bone substitute beyond the demand to simply reduce the harvest of autologous bone.

## 5. Conclusion

The present study examined a possible active bone substitute material by mechanically mixing OCP with autologous bone to fill bone defects. The results suggest that the mixing not only reduces the use of autologous bone from the patient but also activates osteoblastic cellular function and bone regeneration through the bidirectional impact between OCP and autologous bone. Due to experimental limitations regarding the type and amount of autologous bone harvested, further clinically related experiments may establish the clinical relevance of the present materials.
